# Cognitive Control and Bilingualism: The Bilingual Advantage Through the Lens of Dimensional Overlap

**DOI:** 10.3389/fpsyg.2021.614849

**Published:** 2021-02-11

**Authors:** Melodie Bellegarda, Pedro Macizo

**Affiliations:** ^1^Department of Experimental Psychology, University of Granada, Granada, Spain; ^2^Mind, Brain and Behavior Research Center (CIMCYC), University of Granada, Granada, Spain

**Keywords:** bilingual advantage, cognitive control, event-related potentials, Simon task, Stroop task, dimensional overlap

## Abstract

Past research shows that the bilingual experience may enhance cognitive executive function. In this experiment, we evaluated cognitive control in bilinguals relative to monolinguals by using a dimensional overlap model to predict performance in a task composed of Stroop and Simon stimuli. A group of 24 Spanish monolinguals and 24 bilinguals with differing first languages and all having Spanish as a second language (L2) did a picture naming task and a task composed of Stroop and Simon stimuli, where the effect of different overlap conditions (spatial/color) between stimuli and responses were examined. The tasks were performed in Spanish for both groups and performance was indexed with behavioral and electrophysiological measures. We hypothesized that the bilinguals’ daily language practice in L2 reflected overlap conditions similar to the Simon task. Both naming a picture in L2 and the Simon task would involve conflict at the response level. L2 picture naming entails interference between two potential oral responses, to name in L2 vs. L1 (correct vs. incorrect responses, respectively). Similarly, incongruent stimuli in the Simon task produce interference because the irrelevant dimension (spatial location) overlap with an incorrect response. In contrast, the manual Stroop task involves a different type of conflict between two overlapping stimulus dimensions (the ink color and the color meaning). We predicted for these reasons a superior performance in Simon tasks over Stroop tasks for bilinguals, while monolinguals were expected to have a similar performance in both tasks. We also expected to see a correlation between the performance on the picture naming task and the Simon task in bilinguals. However, the behavioral results did not confirm these hypotheses. In fact, both groups had similar congruency effects as measured by reaction times and error rates, and there was no correlation between the picture naming and Simon task in bilinguals. Despite this, the electrophysiological data suggested a relationship between the picture naming task and the P300 congruency effect in bilinguals. Our findings provide insights into the neurocognitive bases of language and serve as a research avenue for language behaviors in bilinguals.

## Introduction

More investigations every year tackle the topic of bilingualism and the many questions it raises, including one of the most critical issues: whether bilingualism confers an advantage over monolinguals in cognitive control. The inhibition of automatic responses in favor of more adaptive goal-directed behaviors is a process that demands cognitive control (Braver and Barch, 2002). However, cognitive control is a much broader construct that involves working memory and cognitive flexibility, which contribute to an individual’s ability for adaptation in changing environments. For bilinguals, this is particularly important because it allows them to reduce lexical and grammatical interferences between languages and, ultimately, prevents incorrect language use given the relevant linguistic environment. Over time, there is an increase in strength of the cognitive executive function due to the constant practice of bilinguals in linguistic tasks that involve conflict resolution (Heidlmayr et al., 2014). Because there is an overlap between the frontal brain regions implicated in cognitive control of language and general attentional and executive control, it is thought that these shared processes can result in the superior performance of bilinguals compared to monolinguals in the resolution of non-verbal conflict tasks (Bialystok et al., 2008). However, there is discordance in the scientific community and investigations that both support and reject the postulation of a bilingual advantage in such tasks (see van den Noort et al., 2019, for a systematic review). This paper will add to the existing body of research and the quest for an answer.

Stroop and Simon tasks are two types of commonly used non-verbal conflict tasks that present different scenarios in which interfering information must be processed and resolved using cognitive control abilities (Stroop, 1935; Simon and Wolf, 1963). In the classic Stroop task, the name of a color (e.g., red) appears in the ink color of its denoted name (e.g., red ink color) or in a different ink color (e.g., blue ink color), creating a congruent or incongruent situation, respectively; the participant must ignore the word while only responding to the ink color. In the Stroop task, slower reaction times (RTs) and higher error percentage are associated with incongruent trials due to higher conflict delaying the response (Bekçi and Karakaş, 2009). In electrophysiological studies using electroencephalogram (EEG), the conflict created during incongruent trials is reflected as a larger negative amplitude (than the amplitude during congruent trials) of the event-related potential (ERP) component dubbed N400, which occurs from 350 to 500 ms after the stimulus onset (Hanslmayr et al., 2008; Naylor et al., 2012). This ERP component reflects the additional processing needed to detect and resolve the conflicting stimulus meaning. The N400 has been associated primarily with increased activity in the anterior cingulate cortex (ACC) (Hanslmayr et al., 2008; Szücs and Soltész, 2010) and a general frontocentral distribution (Bekçi and Karakaş, 2009). The N2 component, peaking at 200–350 ms, has a frontocentral distribution and has been associated with conflict monitoring as well. In particular, its amplitude has been shown to be modulated by response conflict, with greater negative amplitudes being solicited for incongruent stimuli than congruent stimuli (Kousaie and Phillips, 2012b).

The Simon task presents a different kind of interference and conflict resolution scenario, where a participant is presented with visual non-spatial stimuli associated with a specific leftward or rightward response key. Similarly to the Stroop task, RTs and higher error percentage are associated with incongruent trials, in this case, when the stimulus location does not match the response key location. In studies using EEG, resource allocation in regards to task conflict is associated with a specific ERP component: the P300. This positive component, elicited 250–400 ms after stimulus onset, has a less positive amplitude and later peak latency in the incongruent condition compared to the congruent condition (for a review, see Valle-Inclán, 1996; Zhou et al., 2004; Leuthold, 2011). In the incongruent condition, the P300 component can peak anywhere from 20 to 45 ms later than it does in the congruent condition (Melara et al., 2008; Leuthold, 2011). This delay in the incongruent condition is explained as a marker of perceptual interference; put differently, extracting the relevant details of the stimulus can take longer in these incongruent trials due to a mismatch at the response stage. In this way, the P300 can be thought of as a relative measure of the duration of stimulus evaluation. The P300 component has been associated mainly with a centroparietal electrode distribution (Leuthold, 2011; Castro et al., 2018).

Stroop and Simon tasks, as well as other executive control tasks, have been used on bilingual and monolingual populations to investigate if their performance differs in order to draw conclusions about their cognitive control and their ability to adapt to changing demands. In the next paragraphs, we will give an overview of this previous research.

In only the past 10 years, there has been a linear increase in the number of studies on bilingualism and cognitive control, as seen in the Scopus database: from only 2 articles published in the year 2009 to 46 published in the year 2019. However, these original research papers have contrasting results. For the Simon task, some report a general speed advantage for bilinguals over monolinguals (Bialystok et al., 2004) while others report no significant difference in RTs and accuracy (Guido-Mendes, 2015) and no interaction between group and congruency conditions, even when controlling the bilingual group for native speakers, second language (L2) spoken, and immigration status (Paap and Greenberg, 2013). There has been evidence of protective qualities of bilingualism against cognitive decline in old age: a smaller Simon congruency effect is seen in older bilinguals than age matched monolinguals (Bialystok et al., 2004, 2007, 2008). A possible cognitive control advantage in bilinguals has also be explored using the Stroop task. Coderre et al. (2013) manipulated stimulus onset asynchrony (SOA) of the word and color in a Stroop experiment and found that there were reduced interference effects (incongruent – control trials) for English-Chinese bilinguals when compared to English monolinguals, but not of Chinese-English bilinguals. They also found that L2 proficiency modulated these interference results and highlighted the sensitivity for proficiency. Badzakova-Trajkov (2008) found smaller interference scores as well for Macedonian-English and German-English bilinguals compared to English monolinguals. On the other hand, Kousaie and Phillips (2012a) found that while young bilinguals performed the Stroop task faster than young monolinguals, there wasn’t a specific interaction with the condition (incongruent or congruent conditions) and thus, no specific advantage.

There are several meta-analyses and review papers that have been published to try to make sense of the inconsistencies. In a review article by van den Noort et al. (2019), 46 original studies from 2004 to 2018 concerning bilingualism in different cognitive control tasks (e.g., Simon task and Stroop task, but also Flanker task, Attention Network Task, Wisconsin Card Sorting task, N-back task, digit span task, and dichotic listening task, among others) were analyzed. They found that 54.3% of the studies reported beneficial effects of bilingualism, 28.3% reported mixed results, and 17.4% reported evidence against its existence. In another meta-analysis by Lethonen et al. (2018), 152 studies of bilingualism were considered where experimental tasks fell into 6 different executive domains: inhibitory control, set shifting, monitoring, working memory, attention, and verbal fluency. These authors failed to find systematic evidence for a bilingual advantage, even in studies that included better matched participant groups. There was a small effect size found in the domains of inhibitory control, set shifting, and working memory, which subsequently disappeared when taking into account publication bias.

While it is particularly challenging and premature to conclude that bilinguals profit from their knowledge of two languages, specifically in the domain of executive and inhibition control, EEG studies – though few and far between – can speak to how the temporal course of processing stimuli differs between monolinguals and bilinguals. Kousaie and Phillips (2012b) was one of the first studies to consider the bilingual Stroop and Simon experiment from the electrophysiological lens. Although behaviorally there was no difference in RTs or accuracy between language groups when considering congruency, the electrophysiological data told a different story. In the Stroop task, there was a more negative N2 component for monolinguals than bilinguals in all trial types (congruent and incongruent) which the authors interpreted with two possible explanations: either this greater amplitude suggested a greater conflict monitoring in monolinguals and hence more activation of the ACC, or, the smaller amplitude for the bilinguals was the sign of more efficient monitoring that requires less active conflict monitoring. The P300 component in the Stroop task was smaller in both groups for the incongruent trials, indicating a greater resource allocation for the more difficult condition. The Simon N2 component was not different between congruency conditions and groups, but the P300 component was more positive for monolinguals than bilinguals, indicating that for monolinguals, both trial types were easier than for bilinguals and required less resource allocation.

Coderre and van Heuven (2014) continued investigating down this path, using the SOA Stroop experimental paradigm to acquire information on the temporal course of the neural activity responsible for the reported bilingual advantage. Behaviorally, there were no differences in RTs between English monolinguals Chinese (L1) – English (L2) bilinguals except a smaller interference for the bilinguals in the L2 Stroop, which could have been due to reduced language proficiency and a smaller influence of the distracting word. A general non-conflict specific advantage was found for non-linguistic control trials (a string of percent sign) in bilinguals, reflected by faster reaction times and more negative N400 waveforms. The bilinguals L2 Stroop showed a more sustained N400 component in both SOA conditions for both congruency conditions, although there were not amplitude differences between the monolinguals and the L1 Stroop. The authors explained this as the activation of L2 information for bilinguals being more effortful due to reduced automaticity. Differently, in other studies, there was a reduced effect size for the N400 component (incongruent – congruent trials) in the bilingual group in comparison to monolingual groups, which the authors explained as due to a more efficient inhibition of interfering information and reduced orthographic interference (Heidlmayr et al., 2015).

In an experiment with older English monolinguals and balanced older English-French bilinguals, Kousaie and Phillips (2017) studied their performance on Stroop and Simon tasks. Behaviorally there were no differences between bilinguals and monolinguals except for bilinguals being faster and more accurate in incongruent trials than monolinguals (Stroop task only). The N2 was considered as a measure of conflict monitoring and the P300 as a measure of resource allocation and stimulus evaluation (a smaller P300 amplitude indicates more resources used for a more difficult conflict condition). An earlier peaking N2 component was found during the Stroop task for bilinguals (in comparison to monolinguals), but no amplitude differences between the groups. The Stroop P300 amplitude was larger in bilinguals. Both electrophysiological components reflected the behavioral evidence and suggested an earlier conflict detection and the use of less cognitive resources used to solve interference for bilinguals in comparison to monolinguals. For the Simon task, the N2 was larger for incongruent trials than congruent trials only in bilinguals, yet the amplitudes were larger overall for monolinguals. This suggested that monolinguals were monitoring for conflict at a greater degree than bilinguals. For the P300, the bilinguals had more positive amplitude and earlier peaks than monolinguals, which in all suggested the allocation of fewer resources and faster categorization of stimuli.

As seen by the previously detailed studies, the data on the subject of cognitive control in bilinguals and monolinguals remains inconclusive regarding the existence of a bilingual advantage. In our study, we consider the topic of bilingual cognitive control from a different perspective: using the Kornblum Dimensional Overlap (DO) model. This model allows for the classification of different conflict tasks depending on the set of overlapping characteristics contained in their stimuli and response sets (Kornblum, 1994). According to the author, dimensional overlap refers to the degree in which sets of items are perceptually, structurally, or conceptually similar; moreover, the overlap will affect performance on a conflict task regardless of if the overlapping dimensions are relevant to the task (Kornblum, 1994, p. 130). In all, there are 8 different possible ensembles created by taking into account the dimensional overlap between the stimulus-response (SR) dimensions as well as the stimulus-stimulus (SS) dimensions (Kornblum, 1994, p. 131). We will be focusing on two ensemble types that reflect the Simon task and the manual Stroop task. Using the language of the DO model, the Simon task will be from here on labeled as an SR task, due to the overlap between the irrelevant stimulus dimension (i.e., location of the color) and the response dimension (i.e., location of the response key), while having no overlap between the relevant stimulus dimension (i.e., color) and the response dimension. The manual Stroop task, on the other hand, will be labeled as an SS task, due to the overlap between the two stimuli dimensions (i.e., ink color and color meaning of the word), while having no overlap between the response keys and stimulus dimensions (i.e., location, ink color, and color meaning).

The time course of stimuli processing is dependent on the type of task and the differences in dimensional overlap. When a stimulus is presented, and has SS characteristics of conflicting relevancy to the task, the relevant stimulus dimension must be selected and tagged as such before being passed on to the response stage where it is associated with the appropriate response. The effect of irrelevant stimuli dimensions is thus visible early during the stimulus identification stage, as in SS tasks. For a stimulus that does not have conflicting SS characteristics, the presentation of an element from the stimulus set automatically activates the corresponding element in the response set, regardless of if the element is relevant or not to the correct response (Kornblum, 1994, p. 131). If the automatically activated element in the response set is indeed the correct response, it can be selected for execution. However, if it differs, then the automatically activated response must be aborted and the correct response must be selected instead. The effect of irrelevant stimuli dimensions is thus visible later on in processing, during the response production state, as in SR tasks.

The ensembles created by the DO model, specifically the SR and SS overlap ensembles, can be applied to the linguistic gymnastics bilinguals are faced with at the moment they must speak in L2. When an unbalanced L1-L2 speaker wants to name a picture in L2, from the same perceptual stimulus (the picture), s/he has to avoid giving the dominant response (i.e., the L1 picture name) to produce the correct one (i.e., the L2 picture name). That is, from one perceptual stimulus (i.e., the picture) there would be conflict due to the activation of two possible oral responses (i.e., the names of the picture in L1 and L2). This situation parallels the SR overlap ensemble (i.e., Simon task). When a participant is instructed to respond with the left hand to the green color and with the right hand to the blue color, an incongruent stimulus (e.g., a blue color located on the left) produces conflict at the response level because of the overlap between the stimulus irrelevant dimension (i.e., spatial location of the color on the left) and the incorrect response (i.e., left key) which interferes with the correct response (i.e., blue-right key). Therefore, both the SR task and the L2 picture naming task would involve conflict at the response level. On the contrary, the SS overlap ensemble (e.g., manual Stroop task) would represent a different type of conflict as two dimensions of the same stimulus (the background color and the color meaning of the word) interfere with each other in the incongruent condition because they refer to different colors (e.g., the word BLUE presented in a green background). Table 1 shows the relationship between a picture naming task in L2, an SR overlap task, and an SS overlap task in terms of the DO model.

**TABLE 1:** Taxonomy of ensembles in terms of the Dimensional Overlap Model for the L2 picture naming task, SR overlap task, and SS overlap task used in the current study.

Task	Stimulus	Instruction	Overlapping dimensions			
			Stimulus-Response Overlap		Stimulus-Stimulus Overlap	
			Irrelevant overlap	Relevant overlap	Irrelevant overlap	Relevant overlap
L2 picture naming (SR-like task)	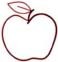	Name the picture in L2	“yes” (strong) picture/incorrect L1 oral response	“yes” (weak) picture/correct L2 oral response	none	none
SR task	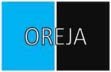	Indicate the stimulus background color: right key – blue left key – green	“yes” color location/response location	“no” color type/response location	“no” color location	“no” color type
SS task	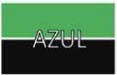	Indicate the stimulus background color: right key – blue left key – green	“no” color meaning/response location	“no” color type/response location	“yes” color meaning “blue”	“yes” color type “green”

*The labels “yes” and “no” refer to whether or not there is overlap between stimulus-response and stimulus-stimulus dimensions according to Kornblum’s (1994) taxonomy for SR and SS tasks. In the SR and SS tasks, the response implies pressing a key spatially located to the right or to the left. In the SR task (Simon task), the relevant dimension of the stimulus (color type) does not overlap with the spatial location of the response (left/right), while the irrelevant dimension (color location) does overlap with the spatial location of the response. In the SS task (manual Stroop task), neither the relevant dimension (color type) nor the irrelevant one (color meaning) overlap with the response location (left/right). However, there is overlap between the relevant and irrelevant dimensions of the stimulus (both the background color and the word meaning refer to colors). In an SR incompatible trial described in the table, conflict occurs at the response level because of the overlap between the stimulus irrelevant dimension (color on the left) and the response (left key) which interferes with the correct response (blue-right key). In an SS incompatible trial described in the table, conflict arises between the dimensions of the stimulus because the irrelevant dimension refers to a different color (blue) than the relevant dimension (green). When an L1 (Spanish) – L2 (English) bilingual has to name a picture in L2, there are two possible responses, to speak in L2 (correct response) or in L1 (incorrect response) from the perceptual stimulus (the picture of an object). For unbalanced bilinguals, the stimulus (the picture of an apple) has a strong irrelevant overlap with the incorrect response because it is spoken in their dominant language (“apple,” the name of the picture in the native language) and a weak relevant overlap with the correct response because it is spoken in their non-dominant language (“manzana,” name of the picture in L2). Thus, in this task, the conflict takes place at the response level (oral production) because of the strong overlap between the stimulus (the picture) and the incorrect response (L1 picture name). Therefore, both the SR task and the L2 picture naming task would involve conflict at the response level.*

If we consider language production for bilinguals in L2 as being a reflection of SR overlap ensembles, it can be said that unbalanced bilinguals actively practice SR overlap tasks everyday simply by communicating in their L2. We hypothesize that this training will reflect on other tasks containing stimulus-response overlaps, such as the Simon task. A bilingual could be expected to perform at a superior level in Simon tasks versus Stroop tasks, seeing that the latter has SS overlapping dimensions which are not related to the overlap faced on a daily basis by bilinguals. Monolinguals should not have this advantage because they do not profit from continuous SR dimensional overlap practice.

The aim of the present study is to use Kornblum’s DO model to investigate cognitive control and bilingualism by looking at differences between performances in conflict tasks in bilinguals and monolinguals. We will use three key tasks: two will be similar to the SR overlap bilinguals are faced with daily (a Simon-like task and a picture naming task in L2) and one will differ (a Stroop-like task containing SS overlap). Using behavioral measures, we propose that bilinguals will perform superiorly in the Simon task in comparison to the Stroop task because their daily cognitive control is reflective of this task. Additionally, we should expect to see a correlation between the performance of bilinguals in the Simon task and the picture naming task. Both these tasks have similar SR overlap, which has been trained in bilinguals. Monolinguals, on the other hand, should perform similarly on both tasks, and we expect no correlations between the picture naming task and the Simon/Stroop task. We plan to take electrophysiological measurements using EEG during the Simon/Stroop task to look at the P300 and N2 components as signs of brain activity regarding resource allocation and conflict monitoring, respectively. Because of the lack of information regarding these components in an interference task such as, it is difficult to predict how the waveforms will be expected to change specifically based on language group. In general, we expect the N2 to have a greater negativity for incongruent trials than congruent trials, reflecting conflict monitoring. This is expected to be reversed for the P300, which has been associated with greater amplitudes for congruent conditions compared to incongruent conditions.

At the time we were writing the research reported here, an article came out (Paap et al., 2019) which is the principal study to use Kornblum’s DO model in regard to bilingual language control. The investigators were searching for an explanation for the superior performance of bilinguals in non-verbal interference tasks, and hypothesized that if SS tasks are resolved using domain general inhibitory control mechanisms, then bilinguals will do better on such tasks than monolinguals. This hypothesis was based on the belief that although both SS and SR competition are faced by bilinguals on a daily basis, SS competition is more prevalent and thus, such similar tasks would confer an advantage. They used English monolinguals and bilinguals with English as a second language (first language was varied) in the experiment, but did not find any evidence of bilinguals having a greater ability to resolve SS conflict tasks.

In contrast to Paap et al. (2019), we will take on a different interpretation of the Kornblum DO taxonomy. We propose that SR tasks will confer a bilingual advantage in the face of SS tasks, following the logic of the dimensional overlaps produced in everyday speech as stated above: when a bilingual speaks, there is competition at the response stage when having to produce a word in their L2. Thus, the SR-like overlap tasks are practiced in bilinguals. We propose to present SS and SR tasks (Stroop and Simon tasks) to a monolingual and a bilingual group, using the same methodology as used by Kornblum (1994). Additionally, we will take the study a step further, recording the electrophysiological activity during the SS/SR tasks to better understand the temporal dynamics at play.

## Materials and Methods

### Participants

Participants were recruited from the University of Granada and the surrounding metropolitan area and in exchange for their participation received economic compensation or course credit toward their university degree. Some participants volunteered for the sake of science, taking part in the experiment without receiving these benefits.

The participants comprised 24 Spanish monolinguals (12 female; mean age = 22.04, *SD* = 3.85) and 24 bilinguals (21 female; mean age = 26.58, *SD* = 7.35) who had Spanish as L2. The selection of participants in each experimental group was done at random. Like in Paap et al. (2019), bilinguals spoke a variety of languages as their mother tongue. There were 12 languages represented in total as first languages, including English (*N* = 5), Basque (*N* = 3), German (*N* = 3), Italian (*N* = 3), Moroccan Arabic (*N* = 3); Hungarian, Japanese, Portuguese, Wolof, Polish, Dutch, and Greek each had one speaker. Participants all had normal vision or corrected to normal vision and there were 43 right-handed participants, 4 left-handed participants, and one ambidextrous participant. Although participants varied in their education background, from vocational training to doctorate degrees there was no significant difference in years of education for the monolingual (*M* years = 17.00, *SD* = 1.769) and bilingual (*M* years = 18.10, *SD* = 2.47) groups, *t*(46) = 1.71, *p* > 0.09. Two participants reported having learning disabilities (ADHD and dyscalculia). The Ethics Committee at the University of Granada approved the experimental procedure used in the study (number issued: 957/CEIH/2019) and each participant provided written informed consent prior to starting the experiment. The ideal sample size was determined using G^∗^Power, version 3.1.9.4 (Faul et al., 2007). It was calculated that for a 2 × 2 × 2 multivariate analysis of variance (MANOVA) to achieve 80% statistical power with α = 0.05 and an effect size of 0.50, the total sample size needed was *N* = 48.

L2 proficiency was evaluated for the bilingual participants using the Peninsular Spanish version of the Language Experience and Proficiency Questionnaire (LEAP-Q) (Marian et al., 2007), in which bilinguals self-reported language history. The different domains assessed in the questionnaire are reported in Table 2. The participants reported having a high overall fluency (understanding, speaking, and reading) of 8.29 out of 10 (*SD* = 1.18). The age of acquisition of 11 of the bilingual participants was from age 3–14 (*M* = 8.36, *SD* = 4.57) and for the remaining was between the ages of 15–29 (*M* = 18.69, *SD* = 3.86).

**TABLE 2:** Self-reported Language Experience and Proficiency Questionnaire (LEAP-Q) for bilingual participants with Spanish as a second language, in reference to their knowledge of Spanish.

	L2 history (Spanish)	
Language history measures	*M*	*SD*
**Self-reported proficiency^a^**		
Understanding	8.33	1.20
Speaking	8.13	1.26
Reading	8.42	1.10
**Age milestones (years)**		
Started learning	13.96	6.67
Attained fluency	19.25	8.49
Started reading	16.88	8.01
Attained reading fluency	20.18	8.16
**Immersion duration (years)**		
In a country	8.00	7.72
In a family	2.13	4.18
In a school/in a place of work	4.79	4.95
**Contribution to language learning^b^**		
Interacting with family	3.50	3.74
Interacting with friends	7.96	2.49
Reading	7.29	2.01
Watching TV	6.21	2.77
Listening to the radio	3.91	3.06
Language tapes/self-instruction	6.33	2.94
**Extent of language exposure^c^**		
Interacting with family	2.58	3.44
Interacting with friends	8.50	2.30
Reading	6.96	2.49
Watching TV	5.71	2.99
Listening to the radio	3.83	3.12
Language tapes/self-instruction	5.33	3.67
**Self-report of foreign accent^d^**		
Perceived by self	4.00	2.57
Perceived by others	5.13	3.75
Present language use (%)	45.39	19.50
Language preference when reading (%)	29.91	24.88
Language preference when speaking (%)	26.08	18.94
Cultural identification with Spanish culture^e^	5.04	3.30

*^a^Range: 0 (none) −10 (perfect). ^b^Range: 0 (not a contributor) – 10 (most important contributor). ^c^Range: 0 (never) – 10 (always). ^d^Range: 0 (none) – 10 (pervasive). ^e^Range: 0 (no identification) – 10 (complete identification). Means (M) and standard deviations (SD) reported.*

### Design and Materials

The experiment was programmed using E-prime 2.0 software (Schneider et al., 2012). The stimuli, data, and analyses of this study are freely available in the Open Science Framework (OSF) repository: https://osf.io/ch684/?view_only=bbfec27395354eb899ef88b356f23f4f

In this study, participants took part in two different tasks, a picture naming task and a task including a combination of different Stroop and Simon stimuli further referred to as the SS/SR task. Each task included first, a training phase to prepare the participants, followed by a task phase in which the experimental data were recorded for later analysis. The SS/SR task has a mixed factorial design of 2 × 2 × 2. Each factor had two levels: Group (monolingual, bilingual), Overlap (SS, SR), and Congruency (congruent, incongruent).

#### Picture Naming Training and Task

The materials consisted of 60 images split evenly between the following 6 categories: four-footed animals, body parts, fruits, kitchen utensils, musical instruments, and vehicles. Pictures were taken from Pérez and Navalón (2003) and Snodgrass and Vanderwart (1980). In the training phase, the participants familiarized themselves with the images and their corresponding Spanish names, viewing each picture for an unlimited duration. During the task phase, the same pictures were presented (without the written name) and participants were instructed to name each image in Spanish out-loud and to be as fast and as accurate as possible. This task was divided into two blocks, with 30 pictures in each block. Each trial consisted of a centrally presented fixation point lasting 1000 ms, followed by an image (281 × 197 pixels) appearing for 500 ms. The next trial did not begin until the participant had responded out-loud to the picture. The pictures were presented in a random order during both the training and the test phase such that no participant received the same order. Response phonation initiation was timed using an Audio-Technica ATR20 Cardioid Low Impedance microphone connected to a PST serial Response Box (Schneider, 1995), and responses were recorded on a Sony ICD PX440 digital voice recorder.

#### SS/SR Training and Task

**FIGURE 1:**
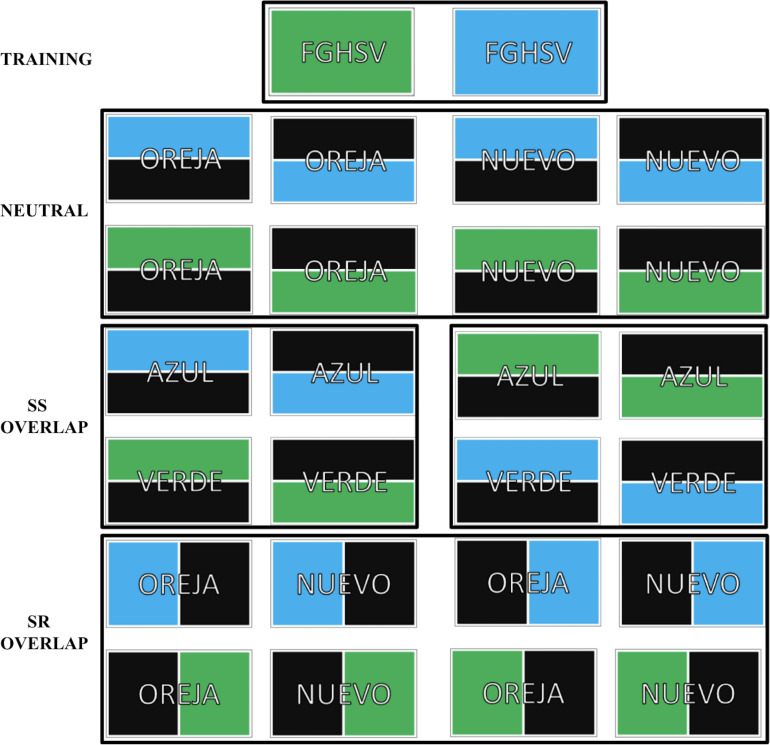
Stimuli for the four possible trial types: Training (2 stimuli), neutral (8 stimuli), SS overlap (8 stimuli), and SR overlap (8 stimuli). Spanish words OREJA, NUEVO, AZUL, VERDE (EAR, NEW, BLUE, GREEN, in English) and the non-word string FGHSV were included.

The stimuli were adapted from Kornblum (1994) and can be referenced in Figure 1. The stimuli consisted of 4 and 5-letter strings surrounded by a rectangle with a colored (blue, green) background. The participants were tasked with responding to the background color using a left or right keystroke (e.g., on a QWERTY layout, the key “Z” for green, the key “M” for blue). The color-key association was counterbalanced between participants.

**FIGURE 2:**
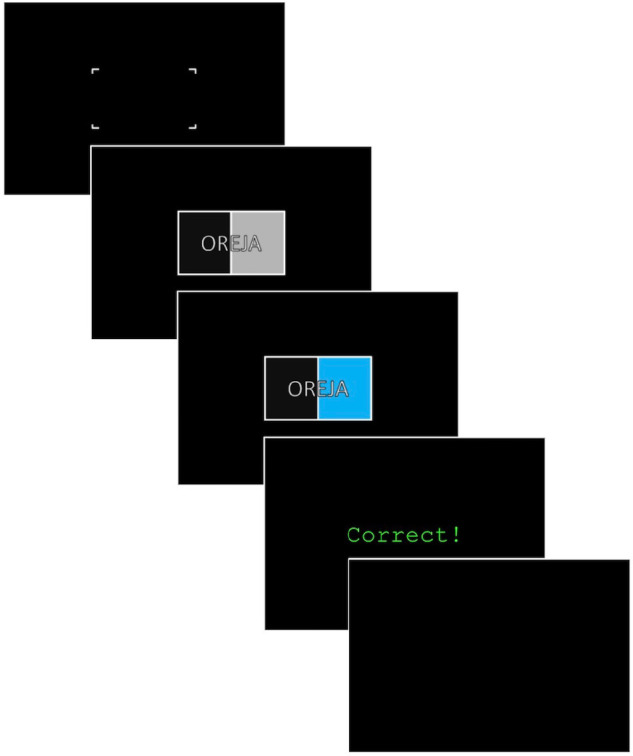
SR/SS trials began with a warning signal (400 ms or 600 ms), followed by a prime (200 ms). The colored stimulus appeared on screen until a key-response was given. Feedback (1500 ms) indicated correctness and accumulated percent accuracy, and a wait period (600 ms or 1200 ms) concluded the trial.

Trial events are noted in Figure 2. After a warning signal (400 ms or 600 ms), a prime appeared for 200 ms until being replaced by the corresponding stimulus. The stimulus presentation was terminated by the participant’s key response. Feedback (1.5 s) indicated correctness (correct or incorrect) and accumulated percent accuracy. A wait period of either 600 ms or 1200 ms concluded the trial.

Participants were first trained for the task with 144 training trials, in which the stimulus string was the non-word “FGHSV” presented on an entirely green or blue background. The SS/SR task phase consisted of 504 trials divided evenly into 7 blocks. Participants were permitted breaks between consecutive blocks. Three different trial types appeared in the SS/SR task: neutral trials, SR overlap trials, and SS overlap trials. Each block included an equal number of each trial type presented in a random order. The stimuli strings in the SS/SR task were Spanish words: OREJA, NUEVO, AZUL, VERDE (EAR, NEW, BLUE, GREEN, in English) superimposed upon a partially colored background. Three different trial types appeared in the SS/SR task: neutral trials, SR overlap trials, and SS overlap trials. In neutral trials, the words OREJA or NUEVO appeared on a partially colored background (upper or lower half). For neutral trials, there was no spatial dimension overlap between the response keys (left or right) and the color location, nor was there a stimulus dimension overlap with the words and background colors presented (the colors blue and green are not associated to OREJA or NUEVO phonetically or semantically). In SS overlap trials, the words AZUL or VERDE appeared on a partially colored background (upper or lower half). For SS overlap trials, there was a stimulus dimension overlap between the words and the background colors presented, which created congruent trials (e.g., the word AZUL is compatible with a blue background) and incongruent trials (e.g., the word AZUL conflicts with a green background). In SR overlap trials, the words OREJA or NUEVO appeared on a partially colored background (left or right half). For SR overlap trials, there was a spatial dimension overlap between the response keys (left or right) and the color location, which created congruent trials (the side of the color and side of the response matched) and incongruent trials (the side of the color and the side of the response conflicted).

A prime, identical to the trial stimulus excepting its gray background color was presented 200 ms before the actual stimulus. The prime gave advance information to the participants by pre-exposing the dimensions of the task which were not relevant to the task, like the word or the position of the color (see Figure 2). Participants were to pay attention only to the color of the stimulus, while ignoring the strings and position of the colored background. The choice to include a prime was based on results from Kornblum (1994), where the author had manipulated the stimulus onset asynchrony of the stimulus components in order to investigate the precise timing of the interference. The author found that a larger congruency effect (as measured by subtracting the RTs of congruent trials from incongruent trials) occurred with a prime 200 ms before the presentation of the stimulus. In our experiment, we aimed at using this design to foster the largest difference between RTs for congruent stimuli and RTs for incongruent stimuli.

### Electrophysiological Recording

After the SS/SR training, the continuous EEG was recorded at a sampling rate of 500 Hz using 64 Ag/AgCl electrodes mounted on a nylon Quik-cap (Compumedics USA, Charlotte, NC, United States) arranged as specified by the extended 10–20 International System (Jasper, 1958). In addition to the 64 scalp electrodes, two electrodes placed above and below the left eye controlled for blinks and two electrodes located near each outer cantus recorded horizontal and vertical eye movements. The electrode impedance was kept below 50kΩ and as close to 5kΩ where possible. Although in the original EEG all channels were referenced against an electrode in the middle of the cap (between Cz and CPz), channels were re-referenced to an average reference offline. The signal was amplified using Neuroscan SynAmps2 amplifiers (El Paso, TX, United States) and was filtered online using a bandpass of 0.01–100 Hz.

SCAN 4.3.1 (Compumedics USA, Charlotte, NC, United States) was used to acquire the EEG. For the offline processing, EEGlab version 2019.0 (Delorme and Makeig, 2004), ERPlab 7.0 (López-Calderón and Luck, 2014) and Matlab R2019a (MATLAB and Statistics Toolbox Release 2019a, The MathWorks, Inc., Natick, MA, United States) were used. This offline processing included applying a low pass 30 Hz filter, correcting for eye blinks and horizontal/vertical eye movements as well as other artifacts during the independent component analysis (ICA). Data was segmented into epochs of [−400 ms 1400 ms], with 0 ms being the digitally tagged time locked presentation of the stimulus. The window of interest of these epochs was taken as [−100 ms 900 ms] to allow us to study the P300 and N200 ERP components. The window [−300 ms −200 ms] was used for baseline correction, taking into consideration the mean activity just before the presentation of the prime from −200 to 0 ms.

### Procedure

The experiment session (lasting approximately 120 min) took place in the EEG recording room where participants were seated at a comfortable distance (70 cm) from a BenQ BL912 48.2 cm Senseye^*TM*^3 LED display monitor which presented stimuli using E-Prime 2.0 software. After a consent form was read and signed by the participant, the following activities took place in the ensuing order: picture naming training and task, SS/SR training and task, and finally, only for those participants forming part of the bilingual group, the Language Experience and Proficiency Questionnaire (LEAP-Q). Together, the picture naming training and task and SS/SR trainings lasted 30 min and the SS/SR task lasted a similar amount of time. The experimenter installed the electrode cap before the SS/SR task, as EEG data was recorded only during that task.

### Statistical Analysis

#### Behavioral

Independent sample *t-*tests were used to test for differences between naming latencies and percent error for both groups in the picture naming task. For the SS/SR behavioral data, the congruency effect (incongruent – congruent condition) was calculated using the response times and error percentages. A mixed two-way ANOVA was calculated for both RTs and percent error, using Group (monolingual, bilingual) as a between-groups factor while Overlap (SR, SS) and Congruency (incongruent, congruent) were within-participants factors.

#### Electrophysiological

For the SS/SR ERP data, the time windows of interest were determined by visually examining the grand averaged waveforms. The N2 component in the SR condition was considered from 280 to 340 ms, the N2 component in the SS condition was considered from 200 to 380 ms, and the P300 components for both SS and SR conditions were considered from 350 to 600 ms. The electrode sites for the analyses were chosen based on the previous literature from Kousaie and Phillips (2017). For the N2 component, levels F1, F3, Fz, F2, and F4 were chosen given previous findings of its relation with a frontocentral distribution; and, for the P300 component, levels C1, C3, Cz, C2, and C4 were chosen based on documented central distribution (Kousaie and Phillips, 2017). The mean of the waveform at each of the five electrode sites (in μVs) was calculated for each participant across the indicated time windows for different overlap (SS, SR) and congruency (congruent, incongruent) conditions. The congruency effect (CE) was calculated as the difference between incongruent and congruent grand mean amplitudes for each overlap condition (SR, SS).

Two general mixed factorial ANOVAs were first performed on all N2 data and P300 data, considering Group (monolingual, bilingual) and Overlap (SS, SR) as between-groups factors while Electrode (F1, F3, Fz, F2, F4 or C1, C3, Cz, C2, C4) and Congruency (incongruent, congruent) were within-participant factors. Four separate ANOVAs were conducted for the N2 SS, N2 SR, P300 SS, and P300 SR data. Group (monolingual, bilingual) was a between-groups factor while Electrode (5 levels – subsequently described) and Congruency (incongruent, congruent) were within-participant factors. In cases where the assumption of sphericity was violated, the Greenhouse and Geisser (G-G) approximation was used (Greenhouse and Geisser, 1959). Original *df*, corrected *p*-values, and the ε correction factors were reported as needed.

#### Correlational Matrices

Two-tailed correlation matrices were calculated for the bilingual group to assess the relationship between the performance (RTs) in the picture naming task and the congruency effects calculated for different measures in the SS/SR task: RTs, N2 grand averages (μVs), and P300 grand averages (μVs). Mean imputation was applied to the bilingual denomination data and EEG data in the case of the missing data points lest the data sets be mismatched sizes.

## Results

We will first report the behavioral results from the picture naming task and the SS/SR task, then electrophysiological results from the SS/SR task, and finally the correlation results between the tasks.

### Picture Naming Task Behavioral Results

Out of the 48 participants, the data from 1 bilingual participant was eliminated from the analysis for the picture naming task due to a computer error. Correct responses for the monolingual and bilingual group were 94.51% (*SE* = 56.15) and 90.94% (*SE* = 53.89), respectively. Values that were 2.5 standard deviations below and above the mean RT of each participant were pruned from the data (5.80% of responses). After this filtering step, the average RT for the monolingual group was calculated to be 812.90 ms (*SD* = 156.30) and there was an average error of 5.49% (*SD* = 4.46). The average response time for the bilingual group was 937.73 ms (*SD* = 154.58) and there was an average error of 9.06% (*SD* = 9.07). Monolinguals took on average 124.8 ms less time to name the pictures than bilinguals, *t*(45) = 2.75, *p* = 0.009, but there was no significant difference in errors made between the two groups, *t*(45) = 1.72, *p* = 0.09.

### SS/SR Task Behavioral Results

**FIGURE 3:**
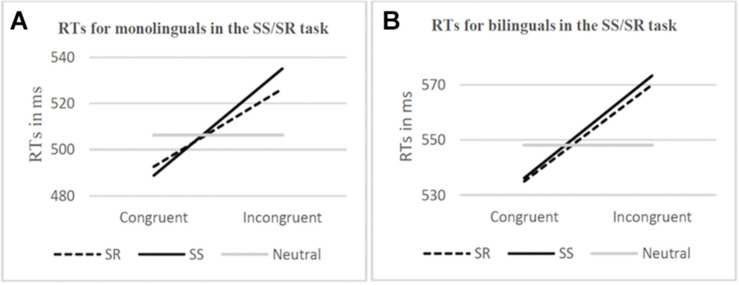
Response times (RTs) [ms] in the SS/SR task for monolinguals **(A)** and bilinguals **(B)**, by Overlap (SR, SS) and Congruency (congruent, incongruent). RTs for neutral stimuli are shown for comparison.

Out of 48 participants, the data from 1 monolingual participant was excluded from the SS/SR task behavioral results analysis due to a computer error when recording. Correct responses for the remaining 47 participants were calculated and were found to be 98.34% (*SE* = 7.87) and 98.19% *(SE* = 13.45) for the monolingual and bilingual groups, respectively. Consistent with Paap et al.’s statistical analysis, response time of less than 200 ms or more than 2.5 standard deviations above the participant’s mean were trimmed (5.62% of responses). The mean reaction time and congruency effect (incongruent condition minus congruent condition) are represented graphically in Figure 3. The congruency effect for the SR task in monolinguals was 33.64 ms and 35.02 ms in bilinguals while the congruency effect for the SS task in monolinguals was 46.21 ms and 37.11 ms in bilinguals. *F*, *p*, and eta squared values are reported from a mixed two-way ANOVA was calculated for both RTs and percent error (see Table 3). Group (monolingual, bilingual) was a between-groups factor while Overlap (SR, SS) and Congruency (incongruent, congruent) were within-participants factors. Response times in the incongruent condition were 38 ms slower on average than in the congruent condition, *F*(1,45) = 65.88, *p* < 0.001 (main effect of Congruency). The Congruency effect was also found in the analysis of the error data, *F*(1,45) = 22.48, *p* < 0.001. All other interactions and effects with RT and error data were not statistically significant.

**TABLE 3:** Mixed 2 × 2 ANOVA results using Congruency (Incongruent, Congruent) and Group (Monolingual, Bilingual) for SS/SR behavioral data.

	RT			% Error		
	*F*(1,45)	*p*	η^2^	*F*(1,45)	*p*	η^2^
**Main effect**						
Group	1.80	0.19	0.04	0.18	0.67	0.004
Overlap	0.62	0.44	0.01	1.12	0.29	0.02
Congruency	65.88	** < 0.001**	0.59	22.48	** < 0.001**	0.33
**Interactions**						
Group * Overlap	0.001	0.97	0.00003	0.21	0.64	0.004
Group * Congruency	0.17	0.68	0.004	0.029	0.96	0.00006
Overlap * Congruency	1.26	0.27	0.03	0.95	0.34	0.02
Group * Overlap * Congruency	0.64	0.43	0.01	0.49	0.49	0.01

In our study, the group of bilinguals was mostly women (87.5%) while the monolingual group was 50% women. It is documented that for women, RTs in behavioral tasks can be significantly prolonged during phases of lower hormone levels (Šimić and Ravlić, 2013). In order to evaluate any gender differences at play in the SS/SR tasks, we considered RT data collected from the female participants in the monolingual (*N* = 11) and bilingual (*N* = 21) groups. At first glance, the female monolingual group followed the trend of faster response times for all conditions, as seen with the original monolingual group. However, a Welch’s *t*-test analysis conducted with RT data revealed that there was no significant difference between the female groups for any of the conditions under study (all *p*s > 0.207). In addition, we randomly sampled the bilingual group to obtain an equally sized comparison groups and found that for the female monolingual (*N* = 11) and bilingual (*N* = 11) groups, there were also no statistically significant differences (all *p*s > 0.804).

### SS/SR Task Electrophysiological Results

Of the 48 participants, the data from 1 participant was excluded due to a computer error when recording, and 3 participants were removed from the analysis due to a high percentage of rejected epochs (> 74%). The final analyses thus considered two language groups comprised of 22 participants each.

**FIGURE 4:**
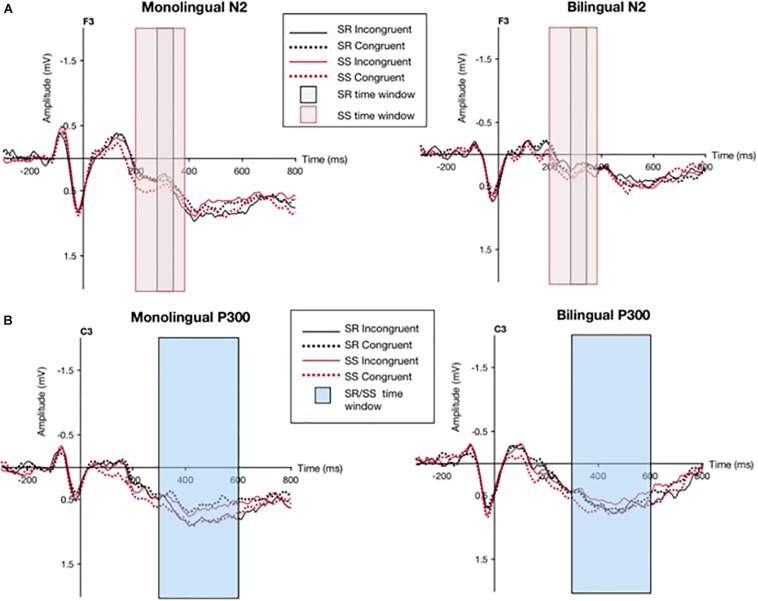
Grand averaged waveform for monolinguals and bilinguals during the different experimental conditions (SR incongruent, SR congruent, SS incongruent, SS congruent) at the F3 and C3 electrode sites. Shaded boxes indicate time window for N2 component **(A)** and P300 component **(B)**.

The grand averaged waveform for monolinguals and bilinguals during the different experimental conditions (SR incongruent, SR congruent, SS incongruent, SS congruent) at the F3 and C3 electrode sites can be seen in Figure 4. Mean amplitudes recorded from each cluster of electrodes for each ERP component across different experimental conditions can be seen in Table 4. For the N2 data, as measured by the average of 5 electrodes (F1, F3, Fz, F2, and F4), the congruency effect for the SR task in monolinguals was −0.09 μV and in bilinguals was 0.07 μV; the congruency effect for the SS task in monolinguals was −0.15 μV and in bilinguals was −0.05 μV. For the P300 data, as measured by the average of 5 electrodes (C1, C3, Cz, C2, and C4), the congruency effect for the SR task in monolinguals was 0.15 μV and in bilinguals was 0.05 μV; the congruency effect for the SS task in monolinguals was 0.04 μV and in bilinguals was −0.09 μV.

**TABLE 4:** Electrophysiological data from SS/SR Task showing grand mean amplitudes (μV) and congruency effects for N2 and P300.

	Monolinguals				Bilinguals			
	N2 [μV] *M* (*SD*)	CE (N2) (I – C)	P300 [μV] *M* (*SD*)	CE(P300) (I – C)	N2 [μV] *M* (*SD*)	CE (N2) (I – C)	P300 [μV] *M* (*SD*)	CE (P300) (I – C)
SR congruent	0.48 (1.87)	−0.09	0.28 (1.04)	0.15	0.27 (0.75)	0.07	0.58 (1.10)	0.05
SR incongruent	0.39 (1.79)		0.43 (1.18)		0.34 (0.81)		0.63 (1.10)	
SS congruent	0.61 (1.91)	−0.15	0.36 (1.15)	0.04	0.38 (0.96)	−0.05	0.63 (1.21)	−0.09
SS incongruent	0.46 (1.95)		0.40 (1.12)		0.33 (0.79)		0.54 (1.19)	

*Measurements are an average of 5 electrodes: F1, F3, Fz, F2, and F4 for the N2 component and C1, C3, Cz, C2, and C4 for the P300 component. The congruency effect (CE) is calculated as the difference between incongruent and congruent grand mean amplitudes (μVs) for each overlap condition (SR, SS).*

For the general mixed factorial ANOVA, a main effect of Electrode was found for the N2 data, *F*(4,340) = 5.84, *p* = 0.004, ε = 0.47, and the P300 data, *F*(4,340) = 3.80, *p* = 0.01, ε = 0.67. There was also an interaction in the P300 data with the factors Congruency x Overlap, *F*(1,85) = 4.29, *p* = 0.04, Electrode x Group, *F*(3,340) = 2.80, *p* = 0.05, ε = 0.67, and Congruency x Electrode x Overlap, *F*(4,340) = 4.42, *p* = 0.005, ε = 0.72.

#### SS Overlap Condition: Stroop Task

The N2 component for the SS Overlap condition was analyzed in a 2 × 2 × 5 ANOVA analysis (Congruency x Group x Electrode) which revealed no significant main effects or interactions. However, both Congruency and Electrode showed a trend toward significance, with values *F*(1,42) = 3.69, *p* = 0.06, and *F*(4,168) = 3.01, *p* = 0.05, ε = 0.50, respectively.

The P300 component for the SS Overlap condition was analyzed in the same way, revealing an interaction for Congruency x Electrode, *F*(4,168) = 3.97, *p* = 0.01, ε = 0.63. All other effects and interactions were not significant. Further analyses from one-way ANOVAs using Congruency (incongruent, congruent) as a repeated measure revealed that this interaction was present in monolinguals only, at the electrode C4, *F*(1,21) = 4.88, *p* = 0.04, η^2^ = 0.18. The interaction between Congruency x Electrode demonstrated a larger mean amplitude for the incongruent condition (*M* = 0.17 μV, *SD* = 1.33) than the congruent condition (*M* = 0.005 μV, *SD* = 1.24) for the C4 electrode in the monolingual group.

#### SR Overlap Condition: Simon Task

The N2 component in the SR Overlap condition was analyzed in a 2 × 2 × 5 ANOVA analysis (Congruency x Group x Electrode) which revealed no significant main effects or interactions, although the main effect of Electrode approached significance, *F*(4,168) = 2.86, *p* = 0.06, ε = 0.44.

The analysis of the P300 component for the SR Overlap condition was analyzed in the same way, revealing a main effect for Congruency, *F*(1,42) = 4.61, *p* = 0.03, indicating a general significant smaller amplitude in the congruent condition (*M* = 0.43 μV, *SD* = 1.08) than the incongruent condition (*M* = 0.54 μV, *SD* = 1.14). There was also an interaction for Congruency x Electrode x Group, *F*(4,168) = 0.02, *p* = 0.04, ε = 0.84. Further analyses from one-way ANOVAs using Congruency (incongruent, congruent) as a repeated measure revealed that this interaction was present in monolinguals only at electrode C3, *F*(1,21) = 6.60, *p* = 0.02, η^2^ = 0.24, at site C1, *F*(1,21) = 11.53, *p* = 0.002, η^2^ = 0.35, and at site C2, *F*(1,21) = 5.83, *p* = 0.02, η^2^ = 0.22. Thus, the 3-way interaction demonstrated a smaller amplitude in the congruent condition (*M* = 0.43 μV, *SD* = 0.88) than the incongruent condition (*M* = 0.66 μV, *SD* = 1.04) reflected in the C3, C1, and C2 electrodes for only the monolingual group.

### Correlation Matrices

**FIGURE 5:**
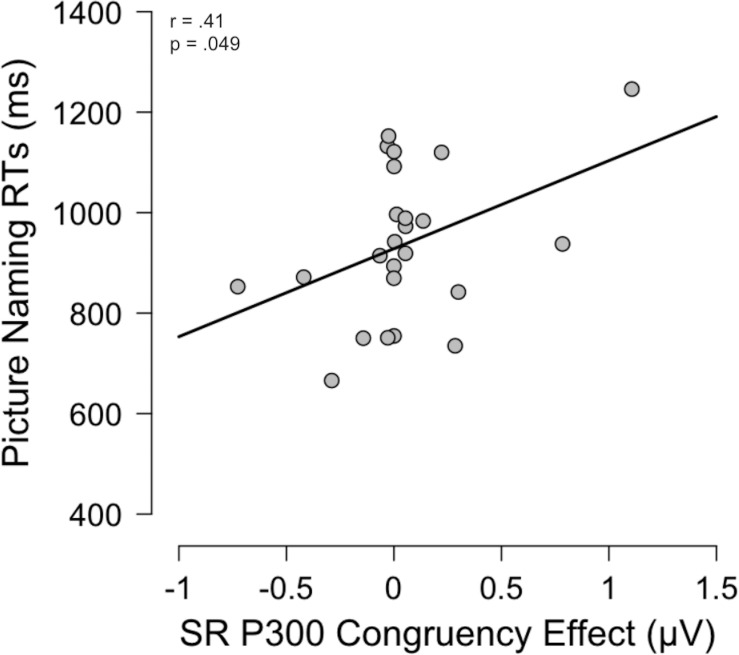
Correlation matrix for picture naming task RTs [ms] and the Congruency effect calculated from the ERP component P300 data in the SR condition [μV].

The data was screened for assumptions and outliers, and no outliers were found. There was a marginal positive correlation between picture naming (RTs) and P300 Congruency effect (incongruent minus congruent condition) in the SR task, *r*(46) = 0.41, *p* = *0.049* (see Figure 5 for a graphical representation of the correlation). The other SR correlations did not yield significant effects: picture naming and SR behavioral congruency effect (RTs) were not correlated, *r*(46) = 0.16, *p* = 0.44, nor were picture naming and N2 congruency effect, *r*(46) = 0.23, *p* = 0.27. For the SS task, neither of the 3 matrices exhibited correlations: picture naming was not correlated with SS behavioral congruency effect, *r*(46) = 0.10, *p* = 0.63, nor N2 congruency effect, *r*(46) = 0.08, *p* = 0.70, nor P300 congruency effect, *r*(46) = 0.13, *p* = 0.56.

## Discussion

The present experiment lies on the foundations of Kornblum DO taxonomy (Kornblum, 1994), which examines compatibility tasks such as Simon and Stroop to understand how dimensional overlap and dimensional relevance affect performance. As the original experiment had been done with monolinguals, the goal of this study was to consider the model in the context of bilingualism (which had been done only recently by Paap et al., 2019) and see if our findings upheld previous investigations demonstrating a bilingual advantage in non-verbal cognitive control tasks. We hypothesized that the cognitive control exerted by bilinguals in a daily setting would give them an upper hand in similar tasks (such as the Simon task) in comparison to dissimilar tasks (such as the manual Stroop task). We also expected to see a correlation between response times in the SR condition and in the picture naming task for bilinguals. Monolinguals, who do not daily practice cognitive control in a an L2 setting, were not expected to perform differently between the Simon and Stroop tasks. Behavioral measures (RTs and error percentages) and electrophysiological measures (ERPs) were both used in the analysis of data. For the ERP data, we examined time windows associated with the N2 and P300 components. In previous experiments, the N2 has been a marker of conflict monitoring, where a larger amplitude denotes higher conflict, and the P300 has been a marker of resource allocation, where a larger amplitude denotes few resources allocated and thus, we expected to see a similar pattern with our data (Kousaie and Phillips, 2017).

As expected, the monolinguals performed better on the picture naming task than the bilinguals, averaging 124.8 ms less time in responding to the pictures. For the bilingual group, we expected to see a correlation between the picture naming and the SR tasks. Behaviorally, a correlation between the picture naming and the congruency effect (incongruent trials – congruent trials) from the SR task would have been indicative of a similar processing and confirmation that we had correctly categorized the picture naming task as having SR overlap. While there were no correlations between these behavioral measures, there was a correlation between the P300 congruency effect in the SR task and the picture naming task. This effect was marginal (*p* = 0.049) so we must be cautious in interpreting these results. In general, the P300 waveform was interpreted as an index of resolving spatial conflict during the process of classifying an event, and the incongruent condition, with less positive amplitudes indicating more cognitive resource allocation for the more difficult condition. However, we acknowledge that the sample of participants evaluated in our study was small (*N* = 48). Thus, future research should examine whether this pattern of results is confirmed as the sample of participants increases which, in turn, would give greater statistical power (> 80%) and a greater effect size (> 0.50). Exploring the relationship between the P300 and bilingual picture naming would also help understand such as to what extent the P300 could be a predictor of bilingualism level (low versus high level of fluency).

Like in Kornblum’s original experiment (Kornblum, 1994), behaviorally we found a significant main effect for congruency which was to be expected, where RTs for the incongruent condition were slower (an average of 38 ms) than for the congruent condition across all overlap conditions and groups. The interaction of most interest to the experiment as evidence of a bilingual advantage was expected to be Group x Congruency x Overlap, where we anticipated seeing a significant difference in performance (RTs) between the SS and SR overlap conditions in the Bilingual group, with smaller congruency effect in the SR condition in comparison to the SS condition. However, this was not found to be significant nor were any other interactions or main effects in the behavioral data. The congruency effect was almost identical between the SR (35.02 ms) and SS (37.11 ms) conditions for bilinguals while the gap was slightly bigger but still not significant for the monolingual SR (33.64 ms) and SS (46.21 ms) conditions. Thus, the critical result of our study was finding that the magnitude of the conflict effects in the SR task and the SS task was comparable in the group of bilingual and monolingual participants. This pattern of results failed to replicate the findings of previous papers which had shown smaller congruency effects for bilinguals in the Simon task (Bialystok et al., 2004) and in the Stroop task (Badzakova-Trajkov, 2008; Coderre et al., 2013).

The lack of differences between the SS and SR task for the monolingual and bilingual groups raises questions about the possible differences associated with the linguistic background of individuals (i.e., bilingualism) in terms of the types of conflict covered by the Dimensional Overlap taxonomy (Kornblum, 1994). Linguistic processing and L2 Spanish fluency were important for both the SR and the SS conditions. These conditions contained linguistic information (words that were irrelevant for performing the task) such as the words EAR and NEW in the SR condition and the words BLUE and GREEN in the SS condition. However, the linguistic component played a different role in the SR and SS tasks. While the conflict in the SS condition depended on lexico-semantic processing (the color meaning of the words and the background color of the screen), the conflict in the SR condition derived from the spatial location of the background color and the response keys. Thus, SS conflict depended on linguistic processing but not SR conflict. However, differences in the linguistic component did not modulate conflict resolution since the same pattern of outcomes was observed in the SS and SR task. Furthermore, the degree of conflict interference due to the SS stimuli for bilinguals should be expected to be modulated in terms of L2 fluency. Bilinguals that have low proficiency in L2 would be expected to have small SS congruency effect explained by reduced automaticity of L2 activation. To evaluate this *post hoc* prediction, we conducted additional analyses taking into consideration the age of L2 acquisition of the bilingual participants in our study. The bilinguals that had acquired Spanish before the age of 15 were on average 69.13 ms slower in the incongruent SS task than the bilinguals that had acquired Spanish later on in life, and they had a larger congruency effect (40.83 versus 33.96), however these data points were not significant. Thus, the age of second language acquisition does not seem to determine the way bilinguals experience interference associated with the processing of irrelevant linguistic information (the color meaning of words) in a conflict task (i.e., SS task). Overall, the pattern of behavioral outcomes obtained in this study converges with those reported in previous research showing the lack of differences between monolinguals and bilinguals in cognitive control tasks involving (or not) the processing of linguistic information. For instance, Duñabeitia et al. (2014) showed equal performance of monolinguals and bilinguals in a verbal Stroop task and a non-verbal version of the same task (i.e., number size-congruency task).

Moreover, on average, the bilingual participants were slower than the monolinguals in all the SS/SR tasks (553.61 ms versus 510.67 ms, respectively), although the differences did not reach significance. Even in the neutral trials, the monolinguals had a better performance than bilinguals (506.35 ms versus 548.09 ms, respectively). It is possible that the variability of the two groups influenced these results: while the monolingual group had an equal number of males and females, the bilingual group was heavily skewed toward females (21 females, 3 males). Previous experiments have shown that females tend to have slower reaction times than males, which may be influencing the outcome in this experiment (Der and Deary, 2006). However, additional analyses reported in the behavioral results section revealed no differences due to the gender of the participants in the performance of the SS/SR tasks. Nevertheless, the bilingual sample itself was variable. Although all bilingual participants had Spanish L2 there were 12 languages represented as native languages, each with a different degree of relatedness to Spanish. There were also bilinguals who considered themselves fully fluent and others only moderately fluent. For example, the self-reported LEAP-Q scores for understanding, speaking, and reading Spanish ranged from 6–10, 5–10, and 6–10, respectively, on the scale 0 (none) – 10 (perfect). It must also be noted that self-estimated measures of language skills also have limitations, and can be skewed by distorted memory (Laine and Lehtonen, 2018). For results of high validity and for further investigations, there is also a need to have as little intergroup variability in terms of background variables which are associated with a superior executive function. These variables include not only socioeconomic status, education, and immigration status but also musical training and time spent playing action video games (Valian, 2015). With regard to monolinguals, finding a population of young adults that has not experienced any form of second language input or learning is difficult, given the amount of foreign language exposure in the modern age (e.g., multilingual cities, the Internet, required language classes in grade school).

Valian (2015) hypothesizes that the inconsistencies in behavioral outcomes for bilingual advantage studies with young adults can stem in part from additional cognitive benefits which this population possesses. Young adults are likely to be involved in cognitively challenging activities which given them cognitive benefits that in turn may compete and/or mask effects that stem from bilingualism. In this study, the Stroop and Simon tasks do not measure one singular process (e.g., cognitive control), but also takes into account, for example, visual and semantic processing, working memory, and cognitive flexibility.

The electrophysiological data showed outcomes that were not revealed in the behavioral data. There was a larger amplitude in the P300 SS condition for monolinguals in the incongruent trials in comparison to the congruent trials. In the P300 SR condition in monolinguals, there was a larger amplitude for the incongruent trials than the congruent trials, localized to the C3, C1, and C2 electrodes. The P300 in the past has been interpreted as a measure of resource allocation, where more cognitive resources are needed for more difficult incongruent trials, and less for congruent trials. This is generally reflected as a less positive P300 (Kousaie and Phillips, 2017). Our results are the opposite of this, as we found greater positive amplitudes for incongruent trials in both the SS and SR tasks in monolinguals. Other authors have suggested that the P300 wave is an index of the attention required to extract information from the stimuli (e.g., incongruent conditions require greater attention and produce larger P300 amplitudes than congruent conditions, Sur and Sinha, 2009). Logically, it makes sense that the incongruent trial would require greater attention than a congruent trial to get from the stimulus presentation stage to the response stage with the correct answer for the trial. However, the P300 has been repeatedly reported as smaller in incongruent conditions (Ragot, 1984; Zhou et al., 2004; Melara et al., 2008) so these theoretical interpretations of the P300 amplitude modulations are hard to reconcile. It is relevant to note that presenting a prime 200 ms before the stimulus could be affecting the subsequent waveform such that the activity reflects residual brain activity stemming from the prime. Additionally, the lack of differences for the N2 component between the monolinguals and bilinguals was surprising. We had expected this component to reflect conflict monitoring, which would theoretically be reduced in bilinguals since they have more practice in monitoring for conflict, and should not require a large activation to find a salient conflict. However, the N2 amplitude was not significantly different in between the groups, suggesting a similar conflict monitoring for both.

To conclude, this study investigated the discussion on bilingualism and cognitive control by considering the situation from the point of view of a dimensional model. While the results did not yield the expected results behaviorally, the electrophysiological data showed that monolinguals in particular showed significant differences between congruency conditions in the P300 component. Additionally, there was a correlation between the P300 congruency effect and the picture naming task in bilinguals which can be elaborated on in the future.

## Data Availability Statement

The datasets presented in this study can be found in online repositories. The names of the repository/repositories and accession number(s) can be found below: Open Science Framework (OSF) repository, https://osf.io/ch684/?view_only=c1e3ec3244b743cf9da83c3ab2cd4e2c.

## Ethics Statement

The study was undertaken in accordance with the 1964 Helsinki Declaration and followed the ethical standards delineated by this journal and by the Ethical Committee of the University of Granada (number issued by the Ethical Committee: 957/CEIH/2019). The participants provided their written informed consent to participate in this study.

## Author Contributions

PM: conceptualization, methodology, funding acquisition, writing – reviewing and editing, and supervision. MB: investigation, formal analysis, visualization, and writing – original draft preparation. All authors contributed to the article and approved the submitted version.

## Conflict of Interest

The authors declare that the research was conducted in the absence of any commercial or financial relationships that could be construed as a potential conflict of interest.
